# Taxonomy, virulence genes and antimicrobial resistance of *Aeromonas* isolated from extra-intestinal and intestinal infections

**DOI:** 10.1186/s12879-019-3766-0

**Published:** 2019-02-14

**Authors:** Yanyan Zhou, Li Yu, Zheng Nan, Pingping Zhang, Biao Kan, Donghui Yan, Jianrong Su

**Affiliations:** 1 Center of Clinical Laboratory, Beijing Friendship Hospital, Capital Medical University, Beijing, 100050 China; 2 Beijing Center for Disease Prevention and Control, Beijing, 100013 China; 3 State Key Laboratory for Infectious Disease Prevention and Control; Department of Diarrheal Diseases, Chinese Center for Disease Control and Prevention, National Institute for Communicable Disease Control and Prevention, Beijing, 102206 China

**Keywords:** *Aeromonas*, Multilocus phylogenetic analysis (MLPA), Virulence genes, Multi-drug resistance

## Abstract

**Background:**

Clinical characteristics (taxonomy, virulence genes and antimicrobial resistance ) of *Aeromonas* in isolated from extra-intestinal and intestinal infections were investigated to describe epidemiology, associated virulence factors and optimal therapy options.

**Methods:**

Clinical samples (*n* = 115) of *Aeromonas* were collected from a general hospital in Beijing between the period 2015 and 2017. Taxonomy was investigate by Multilocus phylogenetic analysis (MLPA), 10 putative virulence factors by use of polymerase chain reaction (PCR) and antimicrobial resistance to 15 antibiotics by use of the microbroth dilution method.

**Results:**

The most common species of *Aeromonas* detected in samples of intestinal tract included; *A. caviae* (43.9%), *A. veronii* (35.7%), and *A. dhakensis* (12.2%). Prevalent species of *Aeromonas* collected from extra-intestinal infections included; *A. hydrophila* (29.4%), *A. caviae* (29.4%), and *A. dhakensis* (23.5%). *A. hydrophila* were detected in 1% of stool samples and 29.4% (5/17) of extra-intestinal infections. *A. hydrophila* strains in extra-intestinal infections were related to malignancy. The most common medical conditions among patients with *Aeromonas* infections included malignancy and liver-transplant related cholecystitis. Multiple drug resistance (MDR) was prevalent in extra-intestinal isolates (82.3%, 14/17) and was greater than the prevalence in intestinal isolates (30.6%, 30/98) (*P* < 0.05). Resistant rates of extra-intestinal isolates were 70.6, 35.3, 23.5 and 5.9% for ceftriaxone, ciprofloxacin, gentamicin and imipenem, respectively, and were higher than found in previous studies. Despite differences in the number and type of virulence genes among samples of *Aeromonas*, no significant correlation was found between invasion and virulent genes in intestinal or extra-intestinal infections.

**Conclusions:**

Overall results of this study support a role for *Aeromonas spp.* as a potential causative infectious agent of gastroenteritis, and malignancy, liver cirrhosis, post liver transplantation in immunocompromised patients. *A. hydrophila* was more prevalent in samples of extra-intestinal infections when compared to samples of intestinal infections, and was especially prominent in samples of patients presenting with malignancy. *Aeromonas* isolates from extra-intestinal samples had high rates of drug resistance but 3rd generation cephalosporins, fluoroquinolones and aminoglycosides remain as options to treat severe diarrhea. However, increasing MDR of extra-intestinal infection samples warrants monitoring.

**Electronic supplementary material:**

The online version of this article (10.1186/s12879-019-3766-0) contains supplementary material, which is available to authorized users.

## Background

The genus *Aeromonas* is a common, gram-negative, facultative anaerobe, coccobacillary-to-bacillary bacteria that belongs to *Aeromonadaceae* [1]*.*The genus *Aeromonas* is comprised of mesophiles and psychrophiles which can cause a number of diseases to warm and cold-blooded animals [2]. Recently, mesophilic *Aeromonas* have received increased attention as an emergent agent of foodborne illness [3]. In humans, *Aeromonas* can cause extra-intestinal diseases, especially in immunocompromised individuals, including septicemia, wound infections, urinary tract infections, hepatobiliary tract infections and necrotizing fasciitis [4].

*Aeromonas* have a complex taxonomy and the genus is comprised of over 30 species, however their identification has been limited by use of conventional biochemical identification methods such as matrix-assisted laser desorption/ionization time of flight masss spectrometry (MALDI-TOF MS), and 16S ribosomal ribonucleic acid (rRNA) sequencing [5–8]. To this end, the use of 5 or more housekeeping genes has been demonstrated as an effective approach for multilocus phylogenetic analysis (MLPA) and species identification of *Aeromonas spp.* [5, 9]. In addition, MLPA has been recommended for the verification of taxonomic affiliation by genome sequencing before being submitted to the NCBI database [10]. Current literature indicates that *A. hydrophila*, *A. veronii bv sobria*, and *A. caviae* are responsible for the majority of human infections and clinical isolations [11]. However, caution must be exercised as *A. dhakensis* can be misidentified as *A. hydrophila* by use of some phenotypic methods [12] and MLPA is suggeted for molecular subtyping [13, 14].

*A. dhakensis* was initially described as a *A. hydrophila* subspecies in 2002, and *A. aquariorum* described later, and was recommended to be reclassified as a separate species in 2012 [15].

The pathogenesis of *Aeromonas spp*. involves a series of virulence factors [16]. Haemolytic toxins include: aerolysin-related cytotoxic enterotoxin (Act) [17], heat-labile cytotonic enterotoxin (Alt), heat-stable cytotonic toxins (Ast) [18], hemolysin (HlyA) and aerolysin (AerA) [19]. In addition, the type III secretion system (TTSS) [20], polar flagellum (fla), lateral flagella (laf) [21, 22], elastase (Ela) [23] and lipase (Lip) [24] contribute to the pathogenicity of *Aeromonas*.

Most cases of diarrheal due to *Aeromonads* are self-limiting and treatment with oral or intravenous fluids is effective. However, patients with serious diarrhea or extra-intestinal infection should receive an antimicrobial treatment [2]. Previously, *Aeromonas* has been observed as resistant to ampicillin, while 3rd generation cephalosporin, fluoroquinolone and aminoglycosides demonstrated excellent antimicrobial activity to *Aeromonas* species isolated from clinical sources [14, 25–27]. However, extensive use of antibiotics in aquaculture and human treatment has led to increasing resistance in bacterium to antimicrobial drugs. Therefore it is prudent to monitor the development of antimicrobial resistance in species of *Aeromonas* to common clinical treatment options.

In the presented study, we investigated characteristics of strains of *Aeromonas* isolated from intestinal infections and extra-intestinal infection. Furthermore we evaluated virulence associated genes and antimicrobial resistance of species of *Aeromonas*.

## Materials and methods

### Isolates of *Aeromonas*

Overall, 1286 stool samples were collected from adults over 14 years old presenting with acute diarrhea at a general hospital in Beijng, China, between June and July 2015, 2017. Epidemiology related medical records were completed to assess clinical history and physical fitness of patients (Additional file 1). Samples of Stool were enriched in alkaline peptone water broth (Beijing landbrige, China) for 8 h at 37 °C, and a loop of the resulting mixture was subcultured on a blood agar plate (Oxoid, UK) supplemented with 20% ampicillin (Sigma, USA) for 16–24 h at 37 °C [28]. An oxidase test (BioMerieuX, France) was performed to select the colonies which were different from Enterobacteriaceae. Microorganisms were identified by use of an automatic bacteriologic analyzer (VITEK2 Compact, BioMerieuX, France). *Salmonella spp*, *Shigella spp* and *Vibrio spp* were also detected on a routine basis. simultaneously.

Extra-intestinal infections due to *Aeromonas* were monitored and the strains were isolated between 2015 and 2017. Clinical samples of blood or bile were cultured in a BACTEC FX400 (BD Diagnostic Instrument Systems, USA). Samples positive for *Aeromonas* were simultaneously subcultured on a blood agar plate and a Maconkey agar plate (BioMerieuX, France). Identification of the isolated microorganisms was completed by use of an automatic bacteriologic analyzer (VITEK2 Compact, BioMerieuX, France). Concurrently, medical records of the patients with extra-intestinal infections due to species of *Aeromonas* were reviewed and age, gender, underlying conditions, microbiological findings and outcome were assembled.

Strains were stored in a Luria broth: glycerol mixture (80:20) at − 80 °C until identification was performed.

### Molecular identification and subtyping of *Aeromonas* isolates

Molecular identification and subtyping of *Aeromonas* isolates was completed by use of 16S rRNA sequencing and MLPA. Total chromosomal DNA from *Aeromonas* was prepared by use of the DNA purification kit (Tiangen Biotech, China) as specified by the manufacturer. PCR amplification was performed by use of 2 × Taq PCR MasterMix (Tiangen Biotech, China). Primers synthesis and sequencing of PCR products were conducted (Shanghai Sangon Biotech, China). Due to the limitations of molecular identification by 16S rRNA sequencing, phylogenetic analysis of the seven selected housekeeping genes *gyrB*, *rpoD*, *recA*, *dnaJ*, *gyrA*, *dnaX* and *atpD* was completed to identify strains of *Aeromonas*. Primers [5] used for PCR amplification are provided in Additional file 2. Concatenated 7-gene phylogenetic trees were constructed and compared with representative species by use of MLPA as previously described [5]. Unrooted neighbour-joining phylogenetic trees were prepared by use of MEGA 5.0 software with Bootstrap values calculated by use of 1000 replicates.

### Detection of virulence-associated genes

The presence of 10 genes encoding virulence factors was determined by use of PCR. Primers are listed in Additional file 2, including *alt* [29], *ast* [30], *hlyA*, *aerA, act, ascF-G* of TTSS, *laf* [14], *lip*, *fla*, and *ela* [31]. PCR amplification reactions were performed at a final volume of 40 μl, containing 20 μl of Taq PCR MasterMix (2×), 1 μl 10 μM primer, 1 μl DNA template (~ 30-40 ng), and 17 μl ddH_2_O. Cycling conditions consisted of an initial single cycle at 95 °C for 5 min, followed by 30 cycles of denaturation at 95 °C for 30 s, annealing was completed at 55 °C–60 °C for 30 s, elongation was completed at 72 °C for 1 min and followed by a final cycle at 72 °C for 7 min. The PCR products were sequenced for further confirmation.

### Antibiotic susceptibility test

Antibiotic susceptibility tests were performed by use of the microbroth dilution method according to guidelines of the current Clinical and Laboratory Standards Institute (CLSI). Minimum inhibitory concentrations (MIC) of strains of *Aeromonas* strains to 15 antibiotics were determined and included; gentamycin (GEN), imipenem (IPM), ampicillin (AMP), cefoxitin (FOX), ceftriaxone (CRO), amoxicillin-clavulanate (AMC), nalidixic acid (NAL), ciprofloxacin (CIP), chloramphenicol (CHL), tetracycline (TCY), doxycycline (DOX), azithromycin (AZM), cefepime (FEP), sulfonamides (Sas) and trimethoprim-sulfamethoxazole (SXT). *E. coli* ATCC 25922 was used as the quality-control strain for susceptibility testing.

### Definitions

Multiple drug resistance (MDR) was was defined as acquired non-susceptibility to at least one agent in three or more antimicrobial categories, according the criteria for defining MDR, XDR and PDR in *Enterobacteriaceae* [32].

Intestinal infections related to a strain of *Aeromonas* were diagnosed as patients presenting with acute diarrhea and a sample culture positive for a strain of *Aeromonas*. Extra-intestinal infections related to a strain of *Aeromonas* were diagnosed as patients presenting with inflammation in a region not identified as intestinal and a sample culture positive for a strain of *Aeromonas*.

### Statistical methods

Data were analyzed by use of the x^2^ test and Fisher’s exact test (SPSS 15.0), When *P* < 0.05 results were considered statistically significant.

## Results

### Clinical features

*Aeromonas spp.* were identified as the causative agent of diarrhea in 98 (7.6%) of 1286 patients. Clinical and epidemiological characteristics were shown in Additional file 3. Gender ratio (male: female) was 0.94 (46/49) among 98 patiens presenting with diarrhea caused by strains of *Aeromonas*. Sources of infections were largely unknown and likely originate from contaminated food. However, 3% of patients identified seafood, cooked food or frozen drinks as likely sources. Twenty percent of patients presented with vomiting, 35.8% abdominal pain, 11.6% fever (body temperature ≥ 37.7 °C), and 9.1% had mild dehydration. Approximately 70% of patients presenting with diarrhea caused by a strain of *Aeromonas* had loose stools for ≥3 times per day, 29.4% had watery stools, and 1.0% had mucus-like stool. Erythrocytes and leukocytes were present in 28.4 and 11.6% of samples of stool collected from patients infected by strains of *Aeromonas* when observed by use of high magnification (HP, × 40). In addition, 6.3% of stool samples presented with erythrocytes and leukocytes. Infection of patients by other enteropathogens was observed in three patients (3/98, 3.1%). Combinations of infectious species included; *Salmonella typhimurium* with *A.caviae*, *Vibrio fluvialis* with *A. veronii* and *Vibrio parahaemolyticus* with *A. veronii.*

Between 2015 and 2017, 17 strains of *Aeromonas* causing extra-intestinal infections were identified (Table 1 and Additional file 4). With the excepted for 3 children accepting a liver transplant (age < 4 years), the average age of the 14 patients was 58.5 years old. The gender ratio (male: female) was 1.83 (11/6). None of the 17 patients were ICU admissions nor was there any acute respiratory failure or mortality. Six (35.3%) patients suffered from *Aeromonas*-related cholecystitis following a liver transplant and 6 (35.3%) pantients presented with malignant tumors. Overall, the most common underlying conditions of patients presenting with *Aeromonas* infections were liver transplantation and malignancy (12/17), In addition, patients presenting with *Aeromonas* related infections were associated with increased prevalence of lung cancer in our study.

**Table 1:** Clinical characteristics of 17 patients presenting with extra-intestinal infections likely caused by species of *Aeromonas*

Strains	Species	Disease spectrum	Underlying condition	Polymicrobial infection
BJ127	*A. dhakensis*	Wound infection after cholecystectomy	Liver cirrhosi and Gallbladder cyst	–
BJ069	*A. dhakensis*	Bacteremia	Pneumonia	*Klebsiella pneumoniae*
BJ123	*A. media*	Bacteremia	Pneumonia and uremia	–
BJ015	*A. dhakensis*	Cholecystitis	Post Liver transplantation	*Stenotrophomonas maltophilia*
BJ022	*A. dhakensis*	Cholecystitis	Post Liver transplantation	*Klebsiella pneumoniae*
BJ016	*A. caviae*	Cholecystitis	Post Liver transplantation	*Klebsiella pneumoniae*
BJ093	*A. caviae*	Cholecystitis	Post Liver transplantation	–
BJ128	*A. caviae*	Chronic Cholecystitis	Post Liver transplantation	*Klebsiella pneumoniae*
BJ124	*A. sanarellii*	Cholecystitis	Post Liver transplantation	–
BJ042	*A. caviae*	Urinary infection	Renal tuberculosis	–
BJ043	*A. caviae*	Urinary infection	Renal insufficiency	–
BJ126	*A. veronii*	Wound infection after Rectal cancer radical resection	Rectal cancer and Esophagus cancer	*Proteus mirabilis*
BJ014	*A. hydrophila*	Hydrothorax	Lung cancer	–
BJ017	*A. hydrophila*	Hydrothorax	Lung cancer	–
BJ018	*A. hydrophila*	Wrapped empyema	Lung cancer	–
BJ054	*A. hydrophila*	Hydrothorax	Esophagus cancer	–
BJ125	*A. hydrophila*	Wound infection after mastectomy	Breast cancer	–

Eleven patients presented with monomicrobial related Aeromonas infections, and 6 patients presented with polymicrobial *Aeromonas* infections (Table 1). Of polymicrobial infections, two consisted of *A.caviae* and *Klebsiella pneumoniae*; and two were *A.aquariorum* with *Klebsiella pneumoniae.* One patient presented with *A.aquariorum* and *Stenotrophomonas maltophilia* and one patient with *A.veronii* and *Proteus mirabilis*. *Klebsiella pneumoniae* was the most common combined pathogen (66.7%, 4/6).

### Genotyping of species of *Aeromonas*

**Fig. 1:**
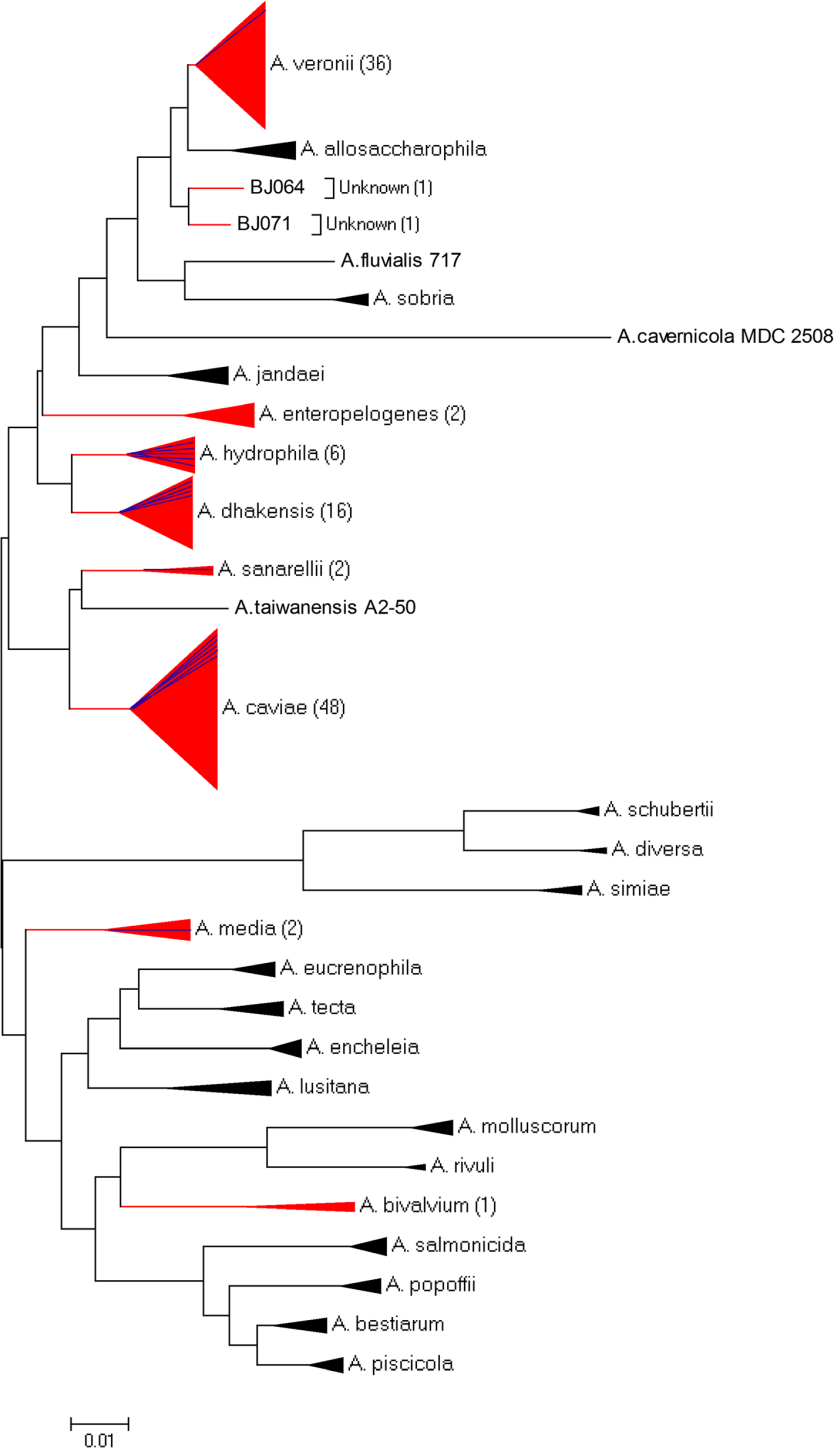
Unrooted neighbor -joined phylogenetic tree of species of *Aeromonas* isolated patients presenting with intestinal or extra-intestinal infections (*n* = 1000 bootstrap replicates). The tree was constructed by use of MLPA of seven housekeeping genes (*gyrB*, *rpoD*, *recA*, *dnaJ*, *gyrA*, *dnaX* and *atpD*). Red tree lines and triangles represent species detected in this study, blue lines represent the numbers of the extra-intestinal infections due to species of *Aeromonas*. The number of identified strains is indicated in brackets. Black tree lines and triangles represent other representative species

Results of MLPA performed with the concatenated 7-gene phylogenetic tree analysis classified 113 of 115 (98.3%) *Aeromonas* isolates to 8 different species (Fig. 1). The four most prevalent species of *Aeromonas* were *A. caviae* (41.7%), *A. veronii* (31.3%), *A. dhakensis* (13.9%), and *A. hydrophila* (5.2%). As presented in Table 2, comparative analysis of genotyping demonstrated differences between intestinal and extra-intestinal isolates was completed. Overall, there was a significant difference in the assemblage of isolates as intestinal isolates generally contained species of *A. caviae* (43.9%), *A. veronii* (35.7%), and *A. dhakensis* (12.2%). In contrast, extra-intestinal isolates generally contained *A. hydrophila* (29.4%), *A. caviae* (29.4%), and *A. dhakensis* (23.5%). There was significant difference between intestinal and extra-intestinal isolates for the species *A. veronii* and *A. hydrophila* (*P* < 0.05, x^2^ test). Five of 6 strains of *A. hydrophila* were isolated from patients with solid tumors, while only 1 strain of *A. hydrophila* was associated with an intestinal infection.

**Table 2:** Distribution of species of *Aeromonas spp.* in isolates collected from intestinal and extra-intestinal samples

Species	No. total strains (%)	No. intestinal isolates (%)	No. extra-intestinal isolates (%)
*A. veronii*	36 (31.3)	35 (35.7)	1 (5.9)
*A. caviae*	48 (41.7)	43 (43.9)	5 (29.4)
*A. dhakensis*	16 (13.9)	12 (12.2)	4 (23.5)
*A. media*	2 (1.7)	1 (1.0)	1 (5.9)
*A. hydrophila*	6 (5.2)	1 (1.0)	5 (29.4)
*A. sanarellii*	2(1.7)	1 (1.0)	1 (5.9)
*A. enteropelogenes*	2 (1.7)	2 (2.0)	0 (0.0)
*A. bivalvium*	1 (0.9)	1 (1.0)	0 (0.0)
Unknown	2 (1.7)	2 (2.0)	0 (0.0)
Total	115	98	17

### Distribution of virulence genes in strains of *Aeromonas*

Presence of multiple virulence genes was common among isolates of *Aeromonas* and 40 virulence combinations of 10 putative virulence genes were identified. The predominant combination (i.e. pattern) of virulence genes was *alt*/*ela*/*lip*/*fla* (pattern1), which presented in 27.0% of patients presenting with an infection related to a isolate of *Aeromonas*. In addition, the patterns of *act*/*fla* (pattern 2), *alt*/*ela*/*lip* (pattern 3) and *act*/*ascF-G*/*fla* (pattern 4) were prevalent among patients*..* As presented in Table 3, the pattern of virulence genes varied among genus’s. Of the four most prevalent species, the haemolytic genes *act* was prevalent in *A. veronii* and *A. dhakensis*. The haemolytic genes *hlyA* was prevalent in *A. hydrophila* and *A. dhakensis*, and the haemolytic genes *aerA* was more prevalent in *A. dhakensis*. The enterotoxin gene *ast* was identified primarily in *A. hydrophila*. The enterotoxin gene *alt*, extracellular protease genes *ela* and *lip* were less prevalent in *A. veronii*. The TTSS genes (*ascF-G*) was prevalent in *A. hydrophila*. *A. veronii* carried pattern 2 and 4 and *A. caviae* carried pattern1 and 3. The species *A. dhakensis* and *A. hydrophila* had diverse virulence patterns, 93.3% *A. dhakensis* and 100% *A. hydrophila* had 5 or more virulence genes.

**Table 3:** Virulence related genes identified in isolates of species of *Aeromonas*

Gene	*A.veronii*	*A. caviae*	*A. dhakensis*	*A. hydrophila*
	No. strains (%)	No. strains (%)	No. strains (%)	No. strains (%)
*ast*	2 (5.6)	0 (0.0)	3 (18.8)	6 (100.0)
*act*	36 (100.0)	1 (2.1)	10 (62.5)	1 (16.7)
*alt*	0 (0.0)	43 (89.6)	14 (87.5)	4 (66.7)
*hlyA*	0 (0.0)	0 (0.0)	14 (87.5)	5 (83.3)
*aerA*	0 (0.0)	0 (0.0)	10 (62.5)	1 (16.7)
*ascF-G*	14 (38.9)	2 (4.2)	4 (25.0)	4 (66.7)
*ela*	6 (16.7)	48 (100.0)	16 (100.0)	6 (100.0)
*lip*	3 (8.3)	48 (100.0)	15 (93.8)	6 (100.0)
*fla*	29 (80.6)	38 (79.2)	16 (100.0)	6 (100.0)
*laf*	2 (5.6)	2 (4.2)	1 (6.3)	2 (33.3)

### Susceptibility to antimicrobials

Resistance profiles of the 115 *Aeromonas* isolates to 15 antimicrobial agents were shown in Table 4. High resistance to ampicillin (93.9%) and Nalidixic acid (54.8%) was observed in *Aeromonas* isolates. The majority of isolates (≥80%) were susceptible to chloramphenicol, gentamicin and the new generation antibiotics ciprofloxacin, ceftriaxone, cefepime, imipenem, sulfonamides, trimethoprim- sulfamethoxazole, doxycycline and azithromycin. Resistance was most prevalent for ciprofloxacin, amoxicillin-clavulanate, cefoxitin, ceftriaxone, sulfonamides, gentamicin and azithromycin in *A. hydrophila*, as a resistance of 66.7, 100.0, 66.7, 66.7, 50.0, 50.0, and 66.7% was observed, respectively. Higher rates of resistance to cefoxitin was also observed in *A. dhakensis* (87.5%). Only 3 strains exhibited resistance to imipenem, all of which were identified as belonging to *A. dhakensis*. Significantly higher rates of resistance to 10 antibiotics (ciprofloxacin, nalidixic acid, amoxicillin-clavulanate, cefoxitin, ceftriaxone, cefepime, sulfonamides, trimethoprim-sulfamethoxazole, gentamicin and azithromycin) were found among extra-intestinal isolates when compared with intestinal isolates (*P* < 0.05, x^2^ test).

**Table 4:** Antibiotic susceptibility patterns of species of *Aeromonas*

	Total			Intestinal infection			Extra-intestinal infection		
Antibiotic	R^a^ [n (%)]	I [n (%)]	S [n (%)]	R [n (%)]	I [n (%)]	S [n (%)]	R [n (%)]	I [n (%)]	S [n (%)]
Ampicillin^c^	108(93.9)	3(2.6)	4(3.5)	92(93.9)	2(2.0)	4(4.1)	16(94.1)	1(5.9)	0(0.0)
Amoxicillin-clavulanate^c^	15(13.0)	76(66.1)	24(20.9)	8(8.2)	68(69.4)	22(22.4)	7(41.2)	8(47.1)	2(11.8)
Imipenem^b^	3(2.6)	10(8.7)	102(88.7)	2(2.0)	7(7.1)	88(89.8)	1(5.9)	3(17.6)	14(82.4)
Ceftriaxone^b^	17(14.8)	3(2.6)	95(82.6)	5(5.1)	3(3.1)	90(91.8)	12(70.6)	0(0.0)	5(29.4)
Cefepime^b^	5(4.3)	3(2.6)	107(93.0)	1(1.0)	0(0.0)	97(99.0)	4(23.5)	3(17.6)	10(58.8)
Cefoxitin^b^	28(24.3)	6(5.2)	81(70.4)	17(17.3)	5(5.1)	76(77.6)	11(64.7)	1(5.9)	5(29.4)
Gentamicin^b^	6(5.2)	4(3.5)	105(91.3)	2(2.0)	2(2.0)	94(95.9)	4(23.5)	2(11.8)	11(64.7)
Nalidixic acid^c^	63(54.8)	–	52(45.2)	49(50.0)	0(0.0)	49(50.0)	14(82.4)	0(0.0)	3(17.6)
Ciprofloxacin^b^	7(6.1)	0(0.0)	108(93.9)	1(1.0)	0(0.0)	97(99.0)	6(35.3)	0(0.0)	11(64.7)
Chloramphenicol^b^	10(8.7)	2(1.7)	103(89.6)	8(8.2)	2(2.0)	88(89.8)	2(11.8)	0(0.0)	15(88.2)
Tetracycline^b^	21(18.3)	5(4.3)	89(77.4)	15(15.3)	3(3.1)	80(81.6)	6(35.3)	2(11.8)	9(52.9)
Doxycycline^c^	4(3.5)	4(3.5)	107(93.0)	2(2.0)	2(2.0)	94(95.9)	2(11.8)	2(11.8)	13(76.5)
Azithromycin^c^	5(4.3)	–	110(95.7)	0(0.0)	–	98(100.0)	5(29.4)	–	12(70.6)
Trimethoprim- Sulfamethoxazole^b^	6(5.2)	–	109(94.8)	1(1.0)	0(0.0)	97(99.0)	5(29.4)	0(0.0)	12(70.6)
Sulfonamides^c^	19 (16.5)	–	96(83.5)	12(12.2)	0(0.0)	86(87.8)	7(41.2)	0(0.0)	10(58.8)

^a^R: Resistant; I: Intermediate; S: Sensitive

^b^Breakpoints are based on the CLSI M45-A3 standards for *Aeromonas spp.*

^c^Other breakpoints refer to the CLSI M100-S26E criteria for *Enterobacteriaceae*

Of the 115 strains, 33 strains (28.7%) exhibited 35 multiple-drug resistance (MDR) patterns to 15 antimicrobial agents. Eighty-three percent (5/6) of strains of *A. hydrophila* and 81.2% (13/16) of strains of *A. dhakensis* presented with MDR, while less MDR isolates were found in *A.caviae* (39.6%, 19/48) and *A.veronii* (16.7%, 6/36). Intestinal strains (30.6%, 30/98) presented with significantly less rates of MDR when compare to isolates from extra-intestinal strains (82.3%, 14/17), indicating acquisition of MDR was likely from in the hospital.

## Discussion

In the presented study, 115 isolates of *Aeromonas* were collected from a general hospital in Beijing between 2015 and 2017. Overall, the abundance and prevalence of strains of *Aeromonas* were different between intestinal and extra-intestinal infections. In our study, 1% of samples isolated from samples of stool of patients with intestinal infection were positive for *A. hydrophila,* while in 29.4% of extra-intestinal infections. Thus, results of this study indicated that the *A. hydrophila* was not the primary pathogen contributing to acute gastroenteritis, however it was more prevalent in extra-intestinal infections when compared to samples from patients with intestinal infections. Interestingly, 5 strains of *A. hydrophila* strains from extra-intestinal infections were present in patients presented with malignant tumor. These results might indicate a preference of strains of *A. hydrophila* and other *Aeromonas spp.* to colonize differently*. A. veronii* was more common in samples of patients presenting with acute gastroenteritis (35.7%) but was rare in patients with extra-intestinal infections (5.9%), which was similar to previous results [14, 33].

In addition, results of our study demonstrate a potential relationship between *Aeromonas* and clinical cirrhosis or malignancy as previously reported [34, 35] and liver-transplant related cholecystitis. These results might be related to bacterial translocation, use of antacids [35] or immunosuppressive agents following liver transplantation.

Prevalence of antimicrobial-resistance was greater in extra-intestinal isolates when compared to the previous study. In our study, rates of resistance to ceftriaxone, ciprofloxacin, gentamicin and imipenem was 70.6, 35.3, 23.5 and 5.9%, while a study completed in Taiwan was 7.7, 6, 3.3 and 1.1%, respectively [36] . Additionally, a study completed in Korea the rates of resistance were 15.5, 10.1, 7.1 and 9.8%, respectively [11]. When compared with the rates of intestinal isolates, the rate of MDR in extra-intestinal isolates was greater. These findings indicate selective pressures in hospitals on strains of infectious bacteria due to the extensive use of antimicrobial agents and warrants more attention in the future.

In our study, two bacteremia-related *Aeromonas* species were identified; *A. media* and *A. dhakensis*. These results were different from previous results where *A. caviae* was identified as bacteremia-related *Aeromonas* species in Japan, *A. hydrophila* and *A. veronii biovar sobria* in Taiwan, and *A. hydrophila* and *A. caviae* in Korea and Taiwan [11, 36].

A study completed in Southern India reported a resistance rate to ceftriaxone resistant of 31% (9/29) for isolates of *Aeromonas* from samples of stool [37]. In our study resistance rates for ceftriaxone, ciprofloxacin and gentamicin and imipenem were 5.1, 1.0, 2.0 and 2.0% in *Aeromonas* isolates of patients presenting with diarrhea and were similar to rates in Shanghai (5.7, 3.6, 0.5, and 2.6%, respectively) [14]. These results along with results of the study competed in Shanghai indicate that 3rd generation cephalosporins, fluoroquinolones and aminoglycosides are a treatment option for severe diarrhea but not for extra-intestinal infections originating in Eastern China.

It is important to note that only 3 strains exhibited resistance to imipenem, all of which belonged to the genus *A. dhakensis*. The genus *A. dhakensis* should be the focus of future research as they harbored high numbers of virulence genes, high rates of drug resistance and a high degree of infection in intestinal and extra-intestinal samples. In addition, *A. hydrophila* presented with a high number of virulence genes and high rates of drug resistance. *A. hydrophila* have previously been isolated from wounds in two cases as reported by Christopher J. Grim et al. [38], and were classified as having MDR and multiple virulence genes.

In the presented study, *Klebsiella pneumoniae* was the most common combined pathogen. These results demonstrate that cholecystitis post Liver transplant predisposed patients to polymicrobial *Aeromonas* infections, while malignant cancers, such as rectal cancer, might predispose patients to monomicrobial *Aeromonas* infection. A previous study in Taiwan found that *E. coli* was the most common pathogen (42%) in polymicrobial infections, then *Klebsiella spp.* (24%) [35]. Conversely, cirrhosis predisposed patients to monomicrobial *Aeromonas* bacteremia while malignant cancer predisposed patients to polymicrobial *Aeromonas* bacteremia [35]. This difference indicates a high degree of heterogeneity in the distribution of intestinal bacteria, and region specific presence of *Aeromonas* infections.

The pathogenic mechanism of *Aeromonas* was multifactorial and complex, and likely involves a series of virulence genes involved in this process. Despite *Aeromonas* harboring different numbers and types of virulence genes, there was no significant correlation found between infection and virulent genes of *Aeromonas* in intestinal infections and extra-intestinal infections. For example, intestinal infections, *alt* have been reported as associated with loose stool, *alt* plus *ast* with watery stools, and *act* with bloody diarrhea [39]. In the presented study, 3 watery stool samples were associated with *ast*, however 25 samples of watery stool were not. In addition, a relationship between infection and presence of virulent genes was not observed and might be related to the limited number of strains isolated in extra-intestinal infections. Similarly, a study completed by Wu et al. found no association between the presence of the genes *aerA, hlyA, alt, ast, ascFG* in isolates of Aeromonas and development of extra-intestinal infections or bacterium [34].

In conclusion, *Aeromonas spp*. should be considered as a causative infectious agent in immunocompromised patients especially those presenting with malignancy, liver cirrhosis and following a liver transplant. In addition, *A. hydrophila* was more prevalent in extra-intestinal infections when compared to intestinal infections, especially for patients presenting with a malignancy. Extra-intestinal *Aeromonas* isolates possessed higher rates of drug resistance*.* However, 3rd generation cephalosporins, fluoroquinolones and aminoglycosides remain as effective treatments for patients presenting with severe diarrhea but not for extra-intestinal infections. In addition, increasing prevalence of drug resistance and MDR in extra-intestinal isolates of *Aeromonas* requires attention and further monitoring.

## Additional files

Additional file 1: Medical Record table upload. (DOCX 14 kb)

Additional file 2: Sequence of primers used for amplification of housekeeping genes and virulence factor genes. (DOC 58 kb)

Additional file 3: Clinical data, genes of toxins and drug resistant patterns for 95 diarrhea patients related to *Aeromonas spp. (XLS 578 kb)*

Additional file 4: Clinical data, genes of toxins and drug resistant patterns for 17 patients related to *Aeromonas spp.* in extra-intestinal infections. (XLS 49 kb)

## Abbreviations

Act: aerolysin-related cytotoxic enterotoxin
AerA: aerolysin
Alt: heat-labile cytotonic enterotoxin
AMC: amoxicillin-clavulanate
AMP: ampicillin
Ast: heat-stable cytotonic toxins
AZM: azithromycin
CHL: chloramphenicol
CIP: ciprofloxacin
CLSI: Clinical and Laboratory Standards Institute
CRO: ceftriaxone
DOX: doxycycline
Ela: elastase
FEP: cefepime
FOX: cefoxitin
GEN: gentamycin
HlyA: hemolysin
HP: high magnification
IPM: imipenem
Lip: lipase
MDR: multiple-drug resistance
MIC: minimum inhibitory concentrations
MLPA: multilocus phylogenetic analysis
NAL: nalidixic acid
PCR: Polymerase chain reaction
Sas: sulfonamides
SXT: trimethoprim-sulfamethoxazole
TCY: tetracycline
TTSS: type III secretion system
