# Supplementary material for: Taxonomy, virulence genes and antimicrobial resistance of Aeromonas isolated from extra-intestinal and intestinal infections
Source: BMC Infect Dis. 2019 Feb 14;19:158. doi: 10.1186/s12879-019-3766-0 (PMC6376669; doi:10.1186/s12879-019-3766-0)
Supplement: Supplementary file 1 — Medical Record table upload. (DOCX 14 kb) [file 12879_2019_3766_MOESM1_ESM.docx]

Medical record table

Name: Gender: Age: Date of birth: Identity number: Native place:

Educational level:

Career: Company/Organization:

Present Address:

phone number:

Date of visit:

Date of onset: Time of onset:

Maximum body temperature:

Abdominal pain: Quadrant of abdomen:

Nausea: Vomiting:

Dehydration status:

Disorder of consciousness: Upper respiratory tract infection:

Systemic poisoning: Shock:

**Routine bloodtest:**

White blood cell count (WBC): Neutrophil count (NEUT#):

Lymphocyte count (LYMPH#): Eosinophil count (EO#):

Basophil count (BASO#):

No. of stools in 24 h:

Stool consistency:  Hematoehezia: Tenesmus:

No. of erythrocytes in stool: No. of leukocytes in stool:

Motile by means of polar flagellum in stool:

Pathogen detection:

Clinical diagnosis:

Patient treatment:

Suspicious food: Way of eating:

Eating Location

Going out of Beijing: Outing area:

Name of Visiting doctor:
