# Supplementary material for: Taxonomy, virulence genes and antimicrobial resistance of Aeromonas isolated from extra-intestinal and intestinal infections
Source: BMC Infect Dis. 2019 Feb 14;19:158. doi: 10.1186/s12879-019-3766-0 (PMC6376669; doi:10.1186/s12879-019-3766-0)
Supplement: Supplementary file 2 — Sequence of primers used for amplification of housekeeping genes and virulence factor genes. (DOC 58 kb) [file 12879_2019_3766_MOESM2_ESM.doc]

**Table S2. Sequence of primers used for amplification of housekeeping genes and virulence factor genes.**

| Locus | Primers | Sequence (5’→3’) | Size of product (bp) | |
| --- | --- | --- | --- | --- |
| 16S rRNA |  |  | |  |
|  | 27F | AGAGTTTGATCCTGGCTCAG | | 1000 |
|  | 1492R | GGYTACCTTGTTACGACTT | |  |
| MLPA |  |  | |  |
| gyrB | gyrB3F | TCCGGCGGTCTGCACGGCGT | | 1126 |
|  | gyrB14R | TTGTCCGGGTTGTACTCGTC | |  |
| rpoD | rpoD4F | GAAGGCGAAATCGACATCGC | | 760 |
|  | rpoD11R | ATGCTCATGCGRCGGTTGAT | |  |
| gyrA | gyrA2F | ATGAGCGATCTGGCCAGAGA | | 815 |
|  | gyrA9R | CGCGCCTTGTTCACCTGATA | |  |
| recA | recAF | VCTVGGTCARATTGAAAAGC | | 849 |
|  | recAR | VTCGCCGTTATAGCTGTACC | |  |
| dnaX | dnaX-1F | CCATACGTTTGAACAAGTGG | | 946 |
|  | dnaX-10R^#^ | AGCTGCACCTCTTCCGG | |  |
| atpD | atpD-0F | CGGCATCAAGGTTATCGACCTG | | 650 |
|  | atpd-7R | ACCAGCGGATCCAGCTGACG | |  |
| dnaJ | dnaJ-F | CGAGATCAAGAAGGCGTACAAG | | 921 |
|  | dnaJ-R | CACCACCTTGCACATCAGATC | |  |
| Virulence |  |  | |  |
|  | astF | ATG CAC GCA CGT ACC GCC AT | | 260 |
| *ast* | astR | ATC CGG TCG TCG CTC TTG GT | |  |
|  | lipF | ATCTTCTCCGACTGGTTCGG | | 382 |
| lip | lipR | CCGTGCCAGGACTGGGTCTT | |  |
|  | elaF | ACACGGTCAAGGAGATCAAC | | 513 |
| ela | elaR | CGCTGGTGTTGGCCAGCAGG | |  |
|  | actF | GAGAAGGTGACCACCAAGAACA | | 232 |
| act | actR | AACTGACATCGGCCTTGAACTC | |  |
|  | altF | GCACGGCGTGACTTCGGTGA | | 576 |
| alt | altR | ACCGCGGTCTTGCAGTTGGG | |  |
|  | aerF | AACCGAACTCTCCAT | | 301 |
| aerA | aerR | CGCCTTGTCCTTGTA | |  |
|  | hlyAF | GGCCGGTGGCCCGAAGATACGGG | | 597 |
| hlyA | hlyAR | GGCGGCGCCGGACGAGACGGG | |  |
|  | flaF | TCCAACCGTYTGACCTC | | 608 |
| fla | flaR | GMYTGGTTGCGRATGGT | |  |
|  | lafF | GGT CTG CGC ATC CAA CTC | | 550 |
| laf | lafR | GCT CCA GAC GGT TGA TG | |  |
|  | ascF-GF | ATG AGG TCATCT GCT CGC GC | | 900 |
| ascF-G | ascF-GR | GGA GCA CAACCA TGG CTG AT | |  |
